# Draft Genome Sequence of Strain B 225, an Iron-Depositing Isolate of the Genus Novosphingobium

**DOI:** 10.1128/genomeA.00325-18

**Published:** 2018-04-12

**Authors:** Burga Braun, Sven Künzel, Ulrich Szewzyk

**Affiliations:** a Technische Universität Berlin, Berlin, Germany; b Max Planck Institute for Evolutionary Biology, Plön, Germany

## Abstract

Here, we report the draft genome sequence of Novosphingobium sp. strain B 225, an iron-depositing bacterium isolated from a phenazone-amended naturally grown biofilm. This biofilm was grown in the Unteres Odertal National Park, Germany. Illumina NextSeq sequencing was used to determine the genome of the strain.

## GENOME ANNOUNCEMENT

Wastewater effluent poses a potential risk to aquatic ecosystems due to persistent organic contaminants which are not eliminated during water treatment. Microbial activity plays a major role in the degradation of environmental pollutants, and members of the genus Novosphingobium have been previously described as contaminant-degrading organisms (1–3).

Based on 16S rRNA gene sequence similarity, strain B 225 belongs to the genus Novosphingobium and shows the highest level of similarity to two Novosphingobium strains, namely, Novosphingobium hassiacum strain W-51 (98%) and Novosphingobium aromaticivorans DSM 12444 (98%), based on BLASTn searches (4), and 97.6% similarity to Novosphingobium ginsenosidimutans by EzBioCloud (5) searches.

The Gram-negative rod-shaped Novosphingobium strain B 225 was isolated from a 4-month-old iron- and manganese-depositing biofilm grown in a continuous-flow biofilm reactor exposed in the Bogengraben River of the Unteres Odertal National Park, Germany. The reactor was continuously fed with river water and phenazone (0.5 g liter^−1^). The biofilm was spread on ATA medium [2 g liter^−1^ MnCO_3_ hydrate, 0.15 g ml^−1^ trisodium citrate, 0.2 g liter^−1^ Fe(NH_4_)_2_(SO_4_)_2_, 0.1 g liter^−1^ cycloheximide, 2 ml vitamin solution (6), 2 ml trace element solution SL 9 (7), 0.94 g phenazone, 20 g agar, and 1 liter filtered water from Lake Daminke] based on Mn agar (8) and incubated for 2 weeks at room temperature. Small dark-brown colonies were selected, and their iron and manganese deposition abilities were confirmed according to Schmidt et al. (9).

Genomic DNA was extracted using the GeneMATRIX soil DNA purification kit (Roboklon, Berlin, Germany). The paired-end library was prepared by using the TruSeq DNA high-throughput (HT) sample prep kit (Illumina Netherlands, Eindhoven, The Netherlands), and mate-pair libraries were established with the Nextera mate-pair sample preparation kit (Illumina Netherlands). The paired-end library was prepared following the Illumina Nextera XT DNA library prep kit protocol. Genome sequencing was done on an Illumina NextSeq 500 sequencer using a NextSeq mid-output kit version 2 with 300-cycle chemistry by generating 10,292,075 raw reads. Demultiplexing was done with bcl2fastq version 2.18.0.12, and quality filtering of raw reads was performed using Trimmomatic version 0.36 (10). The reads were then checked for ambiguous base calls and low complexity, employing the DUST algorithm (11), and filtered accordingly with an R script in Microsoft R Open version 3.3.2 (http://www.r-project.org/), followed by preassembly with SPAdes version 3.10.0 (12) using default k-mer lengths up to 99 bp. Scaffolds ≥500 bp of this preassembly were subject to extension and second-round scaffolding with the SSPACE standard version 3.0 (13). Scaffolds ≥2,500 bp were assigned to genome bins by MetaBAT version 0.32.4 (14), and functional annotation of the draft genomes was performed with Prokka version 1.12b (15).

The draft genome included 73 contigs, with an *N*_50_ assembly quality of 107,389 bp and an *L*_50_ value of 11 bp. The shortest sequence was 2,912 bp, and the longest sequence was 400,854 bp. The total size of the draft genome was 3,712,107 bp, with a GC content of 64%. Annotation included 3,500 coding regions for 3,561 genes, 463 signal peptides, 3 rRNAs (16S and 23S), 49 tRNAs, 1 transfer-messenger RNA (tmRNA), and 8 miscellaneous RNAs (miscRNAs).

### Accession number(s).

This whole-genome shotgun project has been deposited at DDBJ/ENA/GenBank under the accession number MWLM00000000. The version described in this paper is version MWLM01000000.
